# Correlation of Subchondral Bone Density and Structure from Plain Radiographs with Micro Computed Tomography *Ex Vivo*

**DOI:** 10.1007/s10439-015-1452-y

**Published:** 2015-09-14

**Authors:** Jukka Hirvasniemi, Jérôme Thevenot, Harri T. Kokkonen, Mikko A. Finnilä, Mikko S. Venäläinen, Timo Jämsä, Rami K. Korhonen, Juha Töyräs, Simo Saarakkala

**Affiliations:** Research Unit of Medical Imaging, Physics and Technology, Faculty of Medicine, University of Oulu, POB 5000, 90014 Oulu, Finland; Infotech Oulu, University of Oulu, Oulu, Finland; Diagnostic Imaging Center, Kuopio University Hospital, Kuopio, Finland; Department of Applied Physics, University of Eastern Finland, Kuopio, Finland; Medical Research Center Oulu, Oulu University Hospital and University of Oulu, Oulu, Finland; Cancer Center, Kuopio University Hospital, Kuopio, Finland; Department of Diagnostic Radiology, Oulu University Hospital, Oulu, Finland

**Keywords:** Radiography, Micro computed tomography, Bone, Structural analysis, Texture analysis

## Abstract

Osteoarthritis causes changes in the subchondral bone structure and composition. Plain radiography is a cheap, fast, and widely available imaging method. Bone tissue can be well seen from plain radiograph, which however is only a 2D projection of the actual 3D structure. Therefore, the aim was to investigate the relationship between bone density- and structure-related parameters from 2D plain radiograph and 3D bone parameters assessed from micro computed tomography (*µ*CT) *ex vivo*. Right tibiae from eleven cadavers without any diagnosed joint disease were imaged using radiography and with *µ*CT. Bone density- and structure-related parameters were calculated from four different locations from the radiographs of proximal tibia and compared with the volumetric bone microarchitecture from the corresponding regions. Bone density from the plain radiograph was significantly related with the bone volume fraction (*r* = 0.86; *n* = 44; *p* < 0.01). Mean homogeneity index for orientation of local binary patterns (HI_angle,mean_) and fractal dimension of vertical structures (FD_Ver_) were related (*p* < 0.01) with connectivity density (HI_angle,mean_: *r* = −0.73, FD_Ver_: *r* = 0.69) and trabecular separation (HI_angle,mean_: *r* = 0.73, FD_Ver_: *r* = −0.70) when all ROIs were pooled together (*n* = 44). Bone density and structure in tibia from standard clinically available 2D radiographs are significantly correlated with true 3D microstructure of bone.

**Electronic supplementary material:**

The online version of this article (doi:10.1007/s10439-015-1452-y) contains supplementary material, which is available to authorized users.

## Introduction

Plain radiography is a cheap, fast, and widely available imaging method. Especially bone tissue can be well seen from plain radiographs which are significantly contributing the diagnostics of diseases that affect bone density and structure. However, as the plain 2-dimensional (2D) radiograph is a projection (summation) through the actual 3-dimensional (3D) structure, the obtained structural information is always limited compared to true 3D imaging modalities, e.g., computed tomography (CT) and magnetic resonance imaging.

Osteoarthritis (OA) causes changes in the articular cartilage and subchondral bone. Typical OA changes in the subchondral bone include bone sclerosis (thickening), osteophytes, and bone cysts.^6^ Diagnosis of OA is routinely based on clinical examination and changes on plain radiographs. Typically, severity of OA is evaluated from radiographs using Kellgren-Lawrence grading scale.^14^ However, Kellgren-Lawrence grading is based on subjective evaluation, it is semi-quantitative, and its inter-rater and intra-rater reliability varies from moderate to substantial.^11^,^35^,^36^ To exploit all available information from the 2D radiographs, there is a need for development and use of quantitative and user-independent image analysis algorithms.

Previously, quantitative evaluation of OA changes from knee radiographs has included measurement of joint space width^5^,^34^ as well as estimation of bone density^15^,^23^,^42^ and structure.^4^,^17^,^24^–^27^,^40^ It is known that image acquisition parameters and post-processing algorithms affect the bone density evaluation from radiographs.^15^ To overcome this issue, calibration of the grayscale values in an image using an aluminum step wedge have been proposed.^15^,^23^,^42^

Texture analysis of bone is not as dependent on the imaging conditions as the direct evaluation of grayscale values. Bone structural analysis has been performed on plain radiographs using many different algorithms. For instance, the progression of OA has been assessed from digital knee radiography using signature dissimilarity measure method^40^ and fractal signature analysis (FSA).^17^ Fractal-based algorithms have also been applied to macro-radiographs^4^,^24^–^27^ and to standard film radiographs from OA knees.^16^,^31^,^41^ Although macro-radiographs have a better spatial resolution, changes in bone structure can also be detected in standard radiographs.^25^

Recently, local binary patterns (LBP) method for evaluation of bone structure from plain knee radiographs has also been introduced.^12^ Basic LBP methods are computationally efficient and insensitive to monotonic grayscale variations.^28^ Although differences in bone structure between subjects with different stages of OA and controls using LBP-based entropy have been reported,^12^ the LBP-based methods have not been validated against true 3D microarchitecture of bone yet.

Previous studies with bone samples from human cadavers have shown that textural parameters from 2D high-resolution radiographs correlate significantly with 3D trabecular bone parameters.^19^,^29^,^33^,^37^ However, these studies used small specimens harvested from human femur, not the entire bone, and cortical bone was removed from the 3D analyses. Furthermore, image analysis algorithms in these studies were mainly developed for quantification of the osteoporosis-related changes, not specifically for OA-related changes. For example, the fractal parameter (*H*_mean_) that has been used in some osteoporosis-related studies is the average of all possible directions^19^,^29^ while FSA provides fractal signatures in the horizontal and vertical directions.

The degree of relationship between 2D image texture parameters from standard clinical radiographs and 3D micro-CT (*µ*CT) bone parameters from full thickness human tibia is unknown. Therefore, the aim of the current study is to investigate the correlation between bone density- and structure-related parameters from 2D plain radiograph and 3D bone parameters assessed from the *µ*CT scans. We hypothesize that bone density and structure assessed from a 2D image are significantly correlated with the 3D microstructure of bone, possibly providing more sensitive diagnosis of bony changes in OA from conventional radiography.

## Materials and Methods

### Material

Tibial bones from right legs without soft tissue from eleven human cadavers (29–77 years of age) with no history of joint diseases were included in this study.^18^ The cadaver knees were earlier obtained from Jyväskylä Central Hospital, Jyväskylä, Finland, as approved by the national authority (National Authority for Medicolegal Affairs, Helsinki, Finland, Permission 1781/32/200/01).

### Radiography

All bones were imaged using digital radiography (Ysio, Siemens Healthcare, Erlangen, Germany) with constant imaging parameters (63 kVp, 6 mAs, pixel size: 139 × 139 μm^2^, source-detector distance: 151 cm). Subsequently, the bones were immersed into a water bath (radius of the round plastic container: 6 cm) to simulate the effect of soft tissue and imaging was repeated using the aforementioned imaging settings.

### Micro Computed Tomography (*µ*CT)

After the radiography, the bones were cut into halves and both the medial and lateral condyles were imaged with *µ*CT scanner (SkyScan 1176, Bruker MicroCT, Kontich, Belgium, 80 kV, 300 *µ*A, 445 ms exposure, 2 frames averaged, isotropic voxel size of 17.4 μm, 0.04 mm copper + 0.5 mm aluminum filter) separately. Cutting of bones was required to fit the bones into the *µ*CT scanner. To align the *µ*CT slice stacks with the plain radiographs, the μCT slices were manually re-oriented by comparing the 2D coronal projection of the slice stack with the corresponding plain radiograph from the same bone.

For the μCT data, the regions of interest (ROIs) were placed in the 2D coronal projection image from the μCT slices (see: selection of regions-of-interest). After that, ROIs were extracted from every slice of the μCT stack separately. These μCT data, i.e. volumes of interest (VOIs), were then evaluated using SkyScan CTAn software (Bruker MicroCT, Kontich, Belgium). Before calculating the conventional 3D parameters, 3D median filtering (radius 2) and global thresholding (8-bit grayscale value: 95) was applied to extract bone tissue from background. The calculated 3D parameters included bone volume fraction (BV/TV, the ratio of 3D total bone volume to total volume of VOI, in %), average trabecular thickness (Tb.Th, in *µ*m), trabecular separation (Tb.Sp, mean thickness of the non-bone areas, in *µ*m), trabecular number (Tb.N, average number of trabeculae per unit length, in 1/mm), structure model index (SMI, the relative prevalence of rods and plates), and connectivity density (Conn.Dn, the degree of connectivity of trabeculae normalized by total volume of VOI, 1/mm^3^). The definition and calculation of these bone 3D parameters has been thoroughly described in the paper by Bouxsein *et al.*^3^

**Figure 1:**
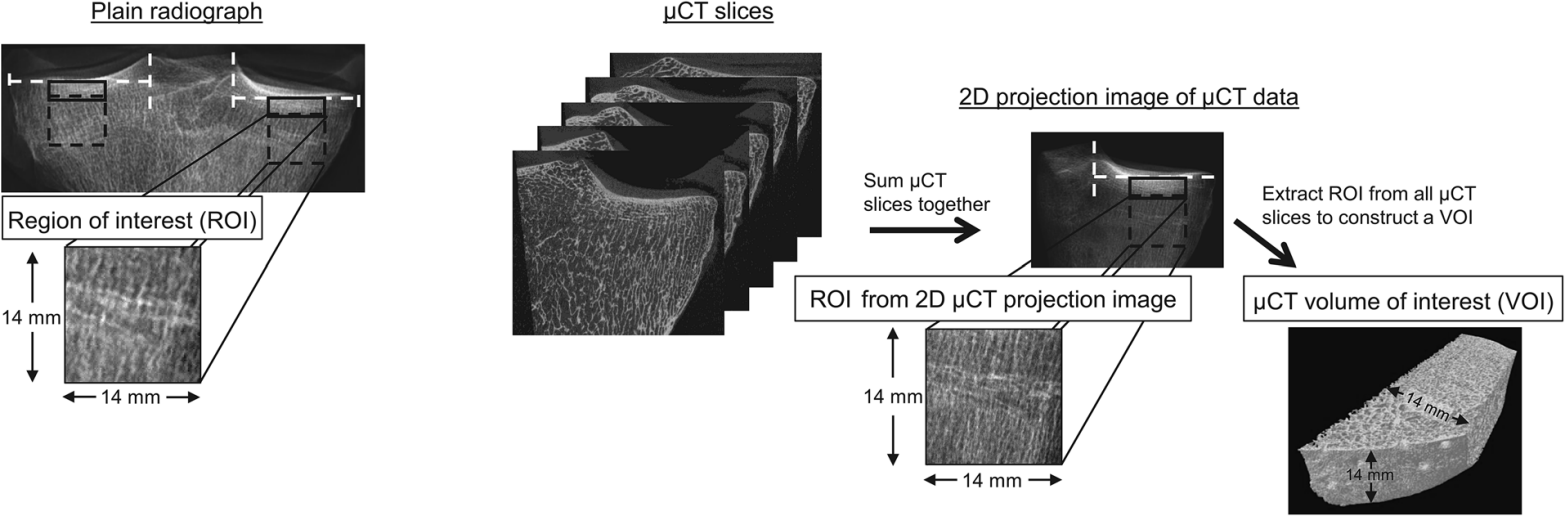
Location of regions of interest (ROIs) and an illustrative presentation how *µ*CT data was processed. Subchondral bone ROIs are marked with continuous black rectangles and trabecular bone ROIs with dashed black colored lines. The purpose of the white dashed lines is to help place the ROIs in the correct locations.

Furthermore, to simulate plain radiography, all binarized μCT slices were summed together to construct a 2D coronal projection image from the 3D μCT data (Fig. 1). This high-resolution 2D projection image was analyzed using the same algorithms as used for plain radiographs to evaluate bone density and structure.

### Selection of Regions of Interest

Four rectangle-shaped ROIs were extracted from the tibia (Fig. 1). Two ROIs (size: 344 × 803 pixels in *µ*CT, 43 × 100 pixels in plain radiographs) were placed into the subchondral bone in the center of the medial and lateral condyles of tibia and two ROIs (803 × 803 pixels in *µ*CT, 100 × 100 pixels in plain radiographs) immediately below the dense subchondral bone in the trabecular bone. Trabecular bone ROIs were aligned horizontally with subchondral bone ROIs. Anatomical landmarks for the ROIs were tibial spine, subchondral bone plate, and outer borders of the proximal tibia. A custom-made MATLAB software (version R2014b, The MathWorks, Inc., Natick, MA, USA) was used for the manual placement of the ROIs.

### Evaluation of Bone Density from Radiographs

To evaluate bone density from the plain radiographs, mean grayscale value of the ROI (=GV) and aluminum step wedge thickness that corresponds to the measured GV (=GV_mmAl_) were determined. The step wedge thickness was calculated using linear interpolation between grayscale values of consecutive steps in the wedge. The step wedge was present in all images.

### Texture Analysis of Bone

Before bone texture analysis, radiographs were median filtered (3 × 3 pixels) to remove high frequency noise from the images. Bone structure was evaluated using Laplacian-based methods,^12^,^39^ LBP-based methods,^12^,^38^ and using FSA.^20^,^21^

#### Laplacian-Based Analysis

Laplacian-based image was constructed as previously described.^12^ The Laplacian-based method enhances the appearance of bone trabeculae and quantifies the variation in the grayscale values of the Laplacian-based image. Laplacians were calculated in the horizontal and vertical directions and summed into one matrix. Subsequently, the unprocessed ROI was multiplied with square root of the Laplacian matrix to enhance the bone and grayscale values were expanded to full dynamic range to obtain the final Laplacian-based image. To measure the randomness of the grayscale values in the Laplacian-based image, entropy of the image (*E*_Lap_) was calculated using the following equation:

1 $$E = - \sum\nolimits_{i} {P_{i} \log_{2} P_{i} }$$

where *P*_*i*_ contains the normalized count of the grayscale value *i* occurring in the image. If *E*_Lap_ = 0, all pixel values in the Laplacian-based image are the same, whereas higher values indicate higher variation in the pixel values of the image.

#### Local Binary Patterns (LBP)-Based Methods

To measure randomness of local patterns and variation in the orientation of adjacent local patterns, LBP-based methods were modified from the methods developed initially for *µ*CT data.^38^ The LBP value of a studied pixel is assessed from the grayscale level of its surrounding, while ignoring the differences in magnitudes. In our method, the image was initially divided into bone and non-bone regions by determining a local threshold for every pixel in the image using Otsu method^30^ with 9 × 9 pixels (36 × 36 for 2D projection image from *µ*CT) window size. Next, LBP operator (8-neighborhood on a circle with a radius of 1) was applied in the bone regions and in the non-bone regions next to bone, i.e. in the bone edge (pixel was considered as an edge pixel if at least one of the 8 neighbors of the center pixel was a bone pixel). To reduce the amount of irrelevant patterns, grouping of patterns was performed by determining the main orientation and the number of valid neighbors (i.e. markers) for each pattern. The main orientation angle was calculated using principal component analysis. The angle (0°, 45°, 90°, and 135°) was calculated only for the patterns that consisted of 2–5 consecutive markers, otherwise the pattern was assigned as non-uniform. Furthermore, to measure the randomness of the patterns occurring in the image, entropy of the grouped patterns (*E*_LBP_) was determined using the Eq. (1). If *E*_LBP_ = 0, there is only single pattern occurring in the image. Finally, the homogeneity index for the orientation of the valid patterns (HI_angle_) was derived from the co-occurrence matrix of the angles. Co-occurrence matrices were calculated in 0°, 45°, 90°, and 135° directions with one pixel distance. The non-uniform and non-bone area was excluded from the co-occurrence matrices. HI at horizontal (${\text{HI}}_{\text{angle}}^{\text{Hor}}$) and vertical (${\text{HI}}_{\text{angle}}^{\text{Ver}}$) directions and mean HI (HI_angle,mean_) of the four possible directions were used in the analyses. If all adjacent patterns have similar orientation, HI_angle_ is one, while a large variation in the orientation of local patterns results a low HI_angle_ value.

#### Fractal Signature Analysis (FSA)

To estimate fractal dimension, related to complexity and roughness of an image, FSA method was used.^20^,^21^ The method produces the fractal signatures in the horizontal and vertical directions at individual scales. In brief, the original 3 × 3 median filtered image was dilated and eroded in horizontal and vertical directions with a rod-shaped one-pixel wide structuring element. The volume, *V*, between dilated and eroded images was then calculated. Calculations were repeated by varying the element length *r* from 2 to 4 pixels. The surface area, *A(r)*, was obtained from the Eq. (2):

2 $$A(r) = {{\left( {V(r) - V(r - 1)} \right)} \mathord{\left/ {\vphantom {{\left( {V(r) - V(r - 1)} \right)} 2}} \right. \kern-0pt} 2}$$

After that a log–log plot was constructed by plotting log of *A*(*r*) against log of *r*. Finally, the fractal dimension was estimated using a regression line to points between 2 and 4 (between 2 and 32 for the 2D projection image from *µ*CT). When the structuring element is pointing in the horizontal direction, fractal dimension of vertical structures (FD_Ver_) is produced and vice versa.^20^ High fractal dimension values are associated with high complexity of the image, whereas low complexity results in low fractal dimension values.

### Statistical Analysis

To evaluate relationships between different parameters, Pearson’s correlation analysis (together with 95% confidence intervals^1^) was applied using IBM SPSS Statistic for Windows (Version 22.0, IBM Corp., Armonk, NY, USA).

## Results

Table 1 shows the mean and standard deviation (SD) values for the 3D *µ*CT parameters. The mean and SD of the 3D *µ*CT parameters in each ROI separately are shown in the Supplementary Table 6.

**Table 1:** Mean and standard deviation (SD) of 3D parameters from *µ*CT (*n* = 44).

3D parameter	Mean ± SD (min–max)
BV/TV (%)	21.8 ± 5.9 (11.8–33.6)
Conn.Dn (1/mm^3^)	6.32 ± 2.04 (3.14–11.51)
Tb.Th (*µ*m)	211 ± 30 (162–283)
Tb.Sp (*µ*m)	723 ± 113 (526–997)
Tb.N (1/mm)	1.02 ± 0.18 (0.73–1.41)
SMI	0.98 ± 0.31 (0.28–1.63)

BV/TV, bone volume fraction; Conn.Dn, connectivity density; Tb.Th, trabecular thickness; Tb.Sp, trabecular separation; Tb.N, trabecular number; SMI, structure model index

### Bone Density

**Figure 2:**
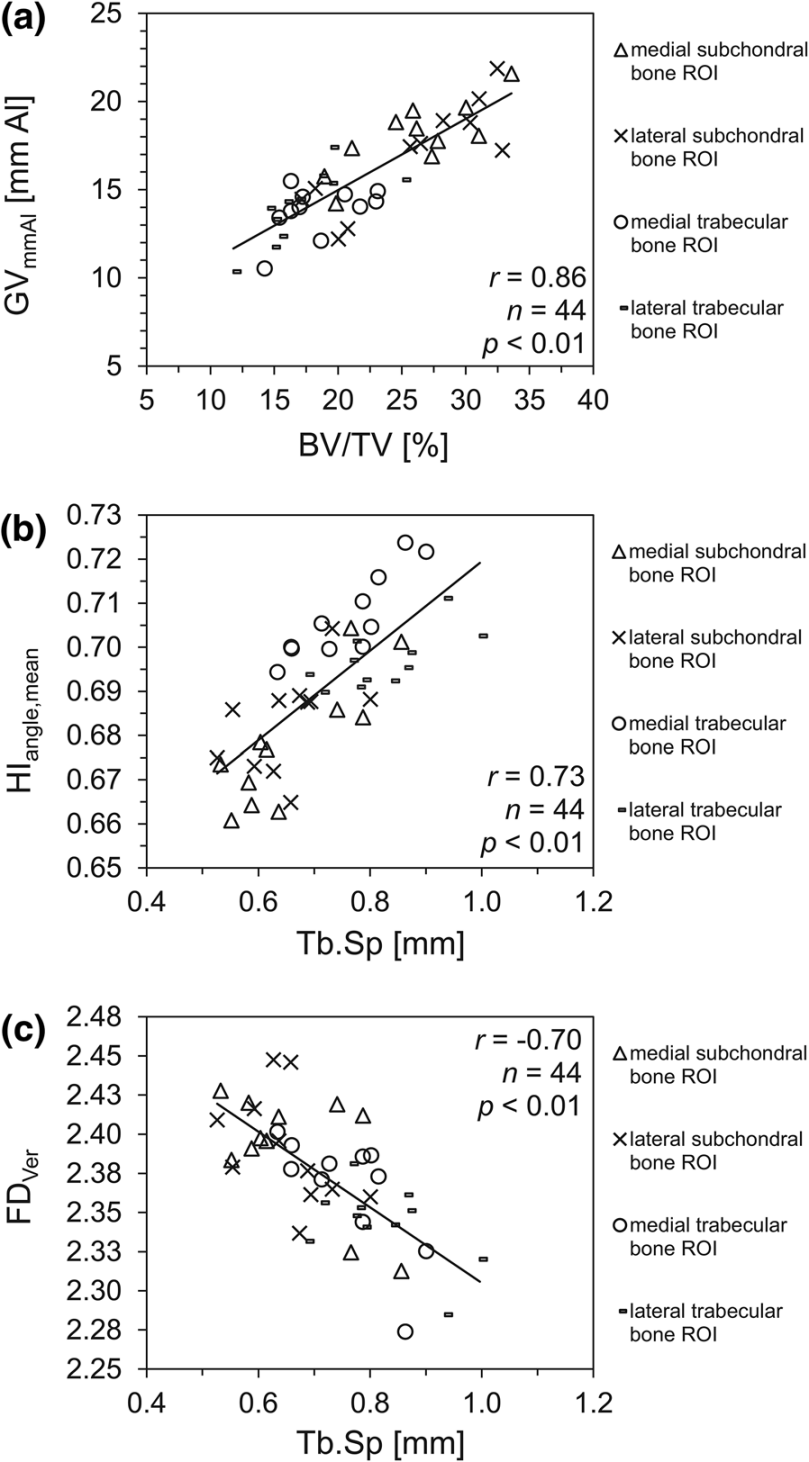
Statistically significant correlations between (a) bone density evaluated from the plain radiograph (GV_mmAl_) and bone volume fraction (BV/TV), (b) mean homogeneity index for orientation of local patterns (HI_angle,mean_) and trabecular separation (Tb.Sp), and (c) fractal dimension of vertical structures (FD_Ver_) and Tb.Sp.

Correlation between bone density evaluated from the plain radiograph using GV_mmAl_ parameter and BV/TV was strong and statistically significant (Table 2; Fig. 2). Correlation remained significant also when the bone was immersed in the water bath during radiography (Table 2). High correlations were observed even when the ROIs were considered separately (Supplementary Table 7).

**Table 2:** Pearson correlation coefficients (95% confidence interval) between bone densities evaluated from both plain radiographs and 2D *µ*CT projection image and BV/TV.

Parameter	BV/TV	BV/TV	BV/TV
	All (*n* = 44)	Subchondral bone (*n* = 22)	Trabecular bone (*n* = 22)
*Plain radiograph*			
GV_mmAl_	0.86** (0.75–0.92)	0.81** (0.58–0.92)	0.61** (0.25–0.82)
GV_mmAl_ (WB)	0.77** (0.61–0.87)	0.70** (0.40–0.87)	0.66** (0.33–0.85)
*µCT 2D projection image*			
GV	0.93** (0.87–0.96)	0.90** (0.76–0.96)	0.86** (0.69–0.94)

**p < 0.01; WB, water bath; GV, mean grayscale value; GV_mmAl_, GV converted to aluminum equivalents

Furthermore, a strong correlation between GV from 2D projection image from *µ*CT data and BV/TV was obtained (Table 2 and Supplementary Table 7).

### Bone Structure

Significant correlations between bone structural parameters from plain radiograph and 3D bone architectural parameters from *µ*CT were obtained, yet, the degrees of correlations varied depending on the parameter and the direction on which the directional texture measures were calculated (Tables 3, 4, 5 and Supplementary Tables 8, 9, 10, and 11). ${\text{HI}}_{\text{angle}}^{\text{Hor}}$ and FD_Ver_ were more strongly related with Conn.Dn and Tb.Sp than ${\text{HI}}_{\text{angle}}^{{{\text{Ve}}r}}$ and FD_Hor_ in the trabecular bone ROIs (Table 5 and Supplementary Tables 10 and 11). In the Fig. 2, significant correlations between HI_angle,mean_ and Tb.Sp and between FD_Ver_ and Tb.Sp are shown.

**Table 3:** Pearson correlation coefficients (95% confidence interval) between bone structure-related parameters from both plain radiographs and 2D *µ*CT projection image and *µ*CT parameters. All ROIs pooled together (*n* = 44).

Parameter	BV/TV	Conn.Dn	Tb.Th	Tb.Sp.	Tb.N.	SMI
*Plain radiograph*						
*E*_Lap_	0.61** (0.38 to 0.77)	0.34* (0.04 to 0.58)	0.59** (0.36 to 0.76)	−0.41** (−0.63 to −0.13)	0.48** (0.22 to 0.68)	−0.51** (−0.70 to −0.25)
*E*_LBP_	0.57** (0.33 to 0.74)	0.61** (0.38 to 0.77)	0.39** (0.11 to 0.62)	−0.57** (−0.74 to −0.32)	0.58** (0.34 to 0.75)	−0.33* (−0.57 to −0.04)
${\text{HI}}_{\text{angle}}^{\text{Hor}}$	−0.13 (−0.41 to 0.17)	−0.52** (−0.71 to −0.26)	0.23 (−0.07 to 0.49)	0.46** (0.18 to 0.66)	−0.37* (−0.60 to −0.09)	−0.11 (−0.39 to −0.20)
${\text{HI}}_{\text{angle}}^{\text{Ver}}$	−0.71** (−0.83 to −0.52)	−0.62** (−0.78 to −0.40)	−0.53** (−0.71 to −0.27)	0.66** (0.46 to 0.80)	−0.67** (−0.81 to −0.46)	0.46** (0.18 to 0.66)
HI_angle,mean_	−0.66** (−0.80 to −0.45)	−0.73** (−0.84 to −0.55)	−0.37* (−0.60 to −0.08)	0.73** (0.56 to 0.85)	−0.71** (−0.83 to −0.53)	0.36* (0.07 to 0.59)
FD_Hor_	−0.04 (−0.33 to 0.26)	0.27 (−0.02 to 0.53)	−0.28 (−0.53 to 0.02)	−0.13 (−0.42 to 0.17)	0.14 (−0.16 to 0.42)	0.09 (−0.21 to 0.38)
FD_Ver_	0.41** (0.12 to 0.63)	0.69** (0.49 to 0.82)	0.03 (−0.27 to 0.32)	0.70** (0.52 to 0.83)	0.60** (0.36 to 0.76)	−0.12 (−0.41 to 0.18)
*E*_Lap_ (WB)	0.35* (0.06 to 0.59)	0.18 (0.06 to 0.59)	0.39** (0.11 to 0.62)	−0.11 (−0.39 to 0.19)	0.25 (−0.05 to 0.51)	−0.30* (−0.55 to −0.01)
*E*_LBP_ (WB)	0.73** (0.55 to 0.84)	0.67** (0.47 to 0.81)	0.44** (0.17 to 0.65)	−0.76** (−0.86 to −0.59)	0.75** (0.58 to 0.86)	−0.48** (−0.68 to −0.21)
${\text{HI}}_{\text{angle}}^{\text{Hor}}$ (WB)	−0.56** (−0.74 to −0.34)	−0.58** (−0.75 to −0.34)	−0.26 (−0.52 to 0.04)	0.64** (0.43 to 0.79)	−0.62** (−0.77 to −0.39)	0.36* (0.07 to 0.60)
${\text{HI}}_{\text{angle}}^{\text{Ver}}$ (WB)	−0.58** (−0.75 to −0.35)	−0.62** (−0.78 to −0.40)	−0.30* (−0.55 to 0.00)	0.64** (0.43 to 0.79)	−0.63** (−0.78 to −0.41)	0.37* (0.08 to 0.60)
HI_angle,mean_ (WB)	−0.59** (−0.75 to −0.34)	−0.64** (−0.79 to −0.42)	−0.29 (−0.54 to 0.01)	0.67** (0.47 to 0.81)	−0.63** (−0.78 to −0.42)	0.34* (0.05 to 0.58)
FD_Hor_ (WB)	0.04 (−0.26 to 0.33)	0.29 (−0.01 to 0.54)	−0.16 (−0.43 to 0.15)	−0.25 (−0.51 to 0.05)	0.20 (−0.10 to 0.47)	0.05 (−0.25 to 0.34)
FD_Ver_ (WB)	0.49** (0.23 to 0.69)	0.76** (0.60 to 0.86)	0.04 (−0.26 to 0.34)	−0.78** (−0.88 to −0.63)	0.70** (0.51 to 0.83)	−0.20 (−0.47 to 0.10)
*2D µCT projection image*						
*E*_Lap_	0.71** (0.53 to 0.83)	0.48** (0.21 to 0.68)	0.64** (0.42 to 0.79)	−0.50** (−0.70 to −0.24)	0.61** (0.38 to 0.77)	−0.58** (−0.75 to −0.34)
*E*_LBP_	0.70** (0.51 to 0.83)	0.72** (0.53 to 0.84)	0.46** (0.19 to 0.66)	−0.71** (−0.83 to −0.52)	0.73** (0.56 to 0.85)	−0.44** (−0.65 to −0.17)
${\text{HI}}_{\text{angle}}^{\text{Hor}}$	−0.70** (−0.82 to −0.50)	−0.80** (−0.89 to −0.66)	−0.36* (−0.59 to −0.07)	0.79** (0.65 to 0.88)	−0.80** (−0.89 to −0.66)	0.41** (0.13 to 0.63)
${\text{HI}}_{\text{angle}}^{\text{Ver}}$	−0.71** (−0.83 to −0.52)	−0.72** (−0.84 to −0.54)	−0.44** (−0.65 to −0.17)	0.72** (0.54 to 0.84)	−0.74** (−0.85 to −0.57)	0.47** (0.20 to 0.67)
HI_angle,mean_	−0.70** (−0.83 to −0.51)	−0.79** (−0.88 to −0.64)	−0.38* (−0.61 to −0.10)	0.78** (0.63 to 0.87)	−0.79** (−0.88 to −0.64)	0.43** (0.15 to 0.64)
FD_Hor_	−0.39** (−0.62 to −0.11)	0.16 (−0.14 to 0.44)	−0.66** (0.45 to 0.80)	0.01 (−0.29 to 0.30)	−0.10 (−0.39 to 0.20)	0.44** (0.17 to 0.65)
FD_Ver_	−0.07 (−0.36 to 0.23)	0.59** (0.36 to 0.75)	−0.55** (−0.73 to −0.30)	−0.46** (−0.67 to −0.19)	0.31* (0.01 to 0.56)	0.31* (0.01 to 0.55)

*p < 0.05, **p < 0.01; WB, water bath; *E*_Lap_, entropy of the Laplacian-based image; FD, fractal dimension of horizontal (Hor) or vertical (Ver) structures; *E*_LBP_, entropy of grouped local binary patterns; HI_angle_, homogeneity index for orientation of local patterns; BV/TV, bone volume fraction; Conn.Dn, connectivity density; Tb.Th, trabecular thickness; Tb.Sp, trabecular separation; Tb.N, trabecular number; SMI, structure model index

**Table 4:** Pearson correlation coefficients (95% confidence interval) between bone structure-related parameters from both plain radiographs and 2D *µ*CT projection image and *µ*CT parameters in subchondral bone ROIs (*n* = 22).

Parameter	BV/TV	Conn.Dn	Tb.Th	Tb.Sp.	Tb.N.	SMI
*Plain radiograph*						
*E*_Lap_	0.61** (0.25 to 0.82)	0.23 (−0.21 to 0.59)	0.53* (0.14 to 0.78)	−0.44* (−0.72 to −0.02)	0.47* (0.06 to 0.74)	−0.48* (−0.75 to −0.07)
*E*_LBP_	0.32 (−0.12 to 0.65)	0.51* (0.11 to 0.77)	0.10 (−0.34 to 0.50)	−0.46* (−0.74 to −0.05)	0.42 (0.00 to 0.71)	−0.09 (−0.49 to 0.35)
${\text{HI}}_{\text{angle}}^{\text{Hor}}$	−0.15 (−0.54 to 0.29)	−0.51* (−0.76 to −0.11)	0.22 (−0.22 to 0.59)	0.46* (0.05 to 0.74)	−0.41 (−0.71 to 0.02)	−0.09 (−0.49 to 0.34)
${\text{HI}}_{\text{angle}}^{\text{Ver}}$	−0.56** (−0.80 to −0.19)	−0.41 (−0.71 to 0.02)	−0.34 (−0.67 to 0.09)	0.64** (0.30 to 0.84)	−0.53* (−0.78 to −0.14)	0.28 (−0.16 to 0.63)
HI_angle,mean_	−0.49* (−0.76 to −0.09)	−0.55** (−0.79 to −0.17)	−0.14 (−0.53 to 0.30)	0.67** (0.35 to 0.85)	−0.59** (−0.81 to −0.23)	0.18 (−0.27 to 0.56)
FD_Hor_	−0.04 (−0.46 to 0.38)	0.36 (−0.07 to 0.68)	−0.37 (−0.68 to 0.06)	−0.18 (−0.56 to 0.26)	0.24 (−0.20 to 0.60)	0.06 (−0.37 to 0.47)
FD_Ver_	0.23 (−0.21 to 0.59)	0.56** (0.18 to 0.79)	−0.14 (−0.53 to 0.30)	−0.54** (−0.78 to −0.15)	0.45* (0.03 to 0.73)	0.01 (−0.42 to 0.42)
*E*_Lap_ (WB)	−0.06 (−0.47 to 0.37)	0.01 (−0.42 to 0.42)	−0.03 (−0.44 to 0.40)	0.21 (−0.24 to 0.58)	−0.03 (−0.45 to 0.40)	−0.01 (−0.43 to 0.41)
*E*_LBP_ (WB)	0.42 (0.01 to 0.71)	0.33 (−0.11 to 0.66)	0.07 (−0.36 to 0.48)	−0.54** (−0.78 to −0.15)	0.46* (0.05 to 0.74)	−0.23 (−0.59 to 0.21)
${\text{HI}}_{\text{angle}}^{\text{Hor}}$ (WB)	−0.43* (−0.72 to −0.01)	−0.26 (−0.61 to 0.18)	−0.09 (−0.50 to 0.34)	0.45* (0.04 to 0.73)	−0.46* (−0.74 to −0.05)	0.37 (−0.06 to 0.68)
${\text{HI}}_{\text{angle}}^{\text{Ver}}$ (WB)	−0.28 (−0.63 to 0.16)	−0.35 (−0.67 to 0.09)	0.07 (−0.36 to 0.48)	0.45* (0.03 to 0.73)	−0.40 (−0.70 to 0.02)	0.17 (−0.27 to 0.55)
HI_angle,mean_ (WB)	−0.31 (−0.65 to 0.13)	−0.33 (−0.66 to 0.11)	0.01 (−0.42 to 0.42)	0.46* (0.05 to 0.74)	−0.39 (−0.70 to 0.04)	0.18 (−0.26 to 0.56)
FD_Hor_ (WB)	−0.03 (−0.45 to 0.39)	0.27 (−0.18 to 0.62)	−0.25 (−0.61 to 0.19)	−0.25 (−0.61 to 0.19)	0.23 (−0.21 to 0.60)	0.10 (−0.34 to 0.50)
FD_Ver_ (WB)	0.16 (−0.28 to 0.55)	0.62** (0.27 to 0.83)	−0.35 (−0.68 to 0.08)	−0.63** (−0.83 to −0.29)	0.53* (0.14 to 0.78)	0.12 (−0.32 to 0.52)
*2D µCT projection image*						
*E*_Lap_	0.57** (0.19 to 0.80)	0.38 (−0.04 to 0.69)	0.32 (−0.12 to 0.65)	−0.56** (−0.80 to −0.19)	0.56** (0.18 to 0.79)	−0.41 (−0.71 to 0.02)
*E*_LBP_	0.68** (0.35 to 0.85)	0.74** (0.46 to 0.89)	0.22 (−0.22 to 0.59)	−0.80** (−0.91 to −0.57)	0.82** (0.60 to 0.92)	−0.40 (−0.70 to 0.02)
${\text{HI}}_{\text{angle}}^{\text{Hor}}$	−0.60** (−0.81 to −0.23)	−0.73** (−0.88 to −0.44)	−0.14 (−0.53 to 0.30)	0.73** (0.44 to 0.88)	−0.77** (−0.90 to −0.51)	0.34 (−0.10 to 0.66)
${\text{HI}}_{\text{angle}}^{\text{Ver}}$	−0.63** (−0.83 to −0.28)	−0.64** (−0.84 to −0.30)	−0.23 (−0.60 to −0.30)	0.70** (0.40 to 0.87)	−0.74** (−0.88 to −0.46)	0.44* (0.02 to 0.73)
HI_angle,mean_	−0.61** (−0.82 to −0.25)	−0.73** (−0.88 to −0.44)	−0.15 (−0.54 to 0.29)	0.74** (0.46 to 0.88)	−0.77** (−0.90 to −0.51)	0.35 (−0.08 to 0.67)
FD_Hor_	−0.26 (−0.61 to 0.18)	0.33 (−0.11 to 0.66)	−0.56** (−0.79 to −0.18)	−0.07 (−0.48 to 0.36)	0.08 (−0.35 to 0.48)	0.23 (−0.21 to 0.59)
FD_Ver_	0.03 (−0.39 to 0.45)	0.79** (0.56 to 0.91)	−0.54** (−0.78 to −0.15)	−0.56** (−0.79 to −0.18)	0.48* (0.08 to 0.75)	0.23 (−0.21 to 0.59)

*p < 0.05, **p < 0.01; WB, water bath; *E*_Lap_, entropy of the Laplacian-based image; FD, fractal dimension of horizontal (Hor) or vertical (Ver) structures; *E*_LBP_, entropy of grouped local binary patterns; HI_angle_, homogeneity index for orientation of local patterns; BV/TV, bone volume fraction; Conn.Dn, connectivity density; Tb.Th, trabecular thickness; Tb.Sp, trabecular separation; Tb.N, trabecular number; SMI, structure model index

**Table 5:** Pearson correlation coefficients (95% confidence interval) between bone structure-related parameters from both plain radiographs and 2D *µ*CT projection image and *µ*CT parameters in trabecular bone ROIs (*n* = 22).

Parameter	BV/TV	Conn.Dn	Tb.Th	Tb.Sp.	Tb.N.	SMI
*Plain radiograph*						
*E*_Lap_	0.16 (−0.28 to 0.54)	−0.11 (−0.51 to 0.33)	0.31 (−0.13 to 0.64)	0.11 (−0.33 to 0.51)	−0.01 (−0.43 to 0.41)	−0.21 (−0.58 to 0.24)
*E*_LBP_	0.04 (−0.39 to 0.46)	0.37 (−0.06 to 0.69)	−0.13 (−0.52 to 0.31)	−0.17 (−0.55 to 0.27)	0.15 (−0.29 to 0.54)	0.11 (−0.32 to 0.51)
${\text{HI}}_{\text{angle}}^{\text{Hor}}$	−0.05 (−0.46 to 0.38)	−0.81** (−0.92 to −0.59)	0.58** (0.21 to 0.81)	0.73** (0.45 to 0.88)	−0.54** (−0.78 to −0.15)	−0.33 (−0.66 to 0.11)
${\text{HI}}_{\text{angle}}^{\text{Ver}}$	−0.04 (−0.45 to 0.39)	−0.36 (−0.68 to 0.08)	0.14 (−0.30 to 0.53)	0.12 (−0.32 to 0.51)	−0.14 (−0.53 to 0.30)	−0.09 (−0.49 to 0.35)
HI_angle,mean_	−0.01 (−0.43 to 0.41)	−0.65** (−0.84 to −0.31)	0.41 (−0.01 to 0.71)	0.42 (0.00 to 0.72)	−0.33 (−0.66 to 0.10)	−0.25 (−0.61 to 0.19)
FD_Hor_	−0.38 (−0.69 to 0.04)	0.06 (−0.37 to 0.47)	−0.47* (−0.74 to −0.06)	−0.01 (−0.43 to 0.41)	−0.16 (−0.54 to 0.28)	0.40 (−0.03 to 0.70)
FD_Ver_	−0.06 (−0.47 to 0.37)	0.65** (0.31 to 0.84)	−0.64** (−0.84 to −0.30)	−0.66** (−0.85 to −0.33)	0.42 (0.00 to 0.72)	0.35 (−0.09 to 0.67)
*E*_Lap_ (WB)	−0.06 (−0.47 to 0.37)	−0.42 (−0.71 to 0.01)	0.32 (−0.12 to 0.65)	0.45* (0.04 to 0.73)	−0.33 (−0.66 to 0.10)	−0.04 (−0.46 to 0.39)
*E*_LBP_ (WB)	0.36 (−0.07 to 0.68)	0.64** (0.30 to 0.84)	−0.02 (−0.44 to 0.41)	−0.54** (−0.79 to −0.16)	0.53* (0.14 to 0.78)	0.01 (−0.42 to 0.42)
${\text{HI}}_{\text{angle}}^{\text{Hor}}$ (WB)	−0.04 (−0.46 to 0.39)	−0.64** (−0.84 to −0.30)	0.33 (−0.11 to 0.66)	0.48* (0.08 to 0.75)	−0.33 (−0.66 to 0.11)	−0.30 (−0.64 to 0.13)
${\text{HI}}_{\text{angle}}^{\text{Ver}}$ (WB)	0.15 (−0.29 to 0.54)	−0.42* (−0.72 to 0.00)	0.37 (−0.06 to 0.69)	0.24 (−0.20 to 0.60)	−0.08 (−0.48 to 0.35)	−0.36 (−0.68 to 0.08)
HI_angle,mean_ (WB)	0.07 (−0.36 to 0.48)	−0.61** (−0.82 to −0.26)	0.41 (−0.01 to 0.71)	0.42 (−0.01 to 0.71)	−0.24 (−0.60 to 0.20)	−0.40 (−0.70 to 0.03)
FD_Hor_ (WB)	−0.38 (−0.69 to 0.05)	0.16 (−0.28 to 0.54)	−0.49* (−0.75 to −0.08)	−0.12 (−0.51 to 0.32)	−0.12 (−0.51 to 0.32)	0.37 (−0.06 to 0.68)
FD_Ver_ (WB)	0.16 (−0.28 to 0.55)	0.72** (0.43 to 0.87)	−0.42 (−0.71 to 0.01)	−0.74** (−0.89 to −0.47)	0.57** (0.20 to 0.80)	0.15 (−0.29 to 0.54)
*2D µCT projection image*						
E_Lap_	0.23 (−0.21 to 0.60)	−0.18 (−0.56 to 0.26)	0.47* (0.07 to 0.75)	0.28 (−0.16 to 0.63)	−0.04 (−0.45 to 0.39)	−0.33 (−0.66 to 0.11)
E_LBP_	0.27 (−0.17 to 0.62)	0.48* (0.08 to 0.75)	0.03 (−0.39 to 0.45)	−0.34 (−0.67 to 0.09)	0.35 (−0.09 to 0.67)	0.02 (−0.41 to 0.43)
${\text{HI}}_{\text{angle}}^{\text{Hor}}$	−0.36 (−0.68 to 0.08)	−0.69** (−0.86 to −0.38)	0.11 (−0.33 to 0.51)	0.65** (0.31 to 0.84)	−0.57** (−0.80 to −0.20)	−0.05 (−0.46 to 0.38)
${\text{HI}}_{\text{angle}}^{\text{Ver}}$	−0.29 (−0.64 to 0.15)	−0.53* (−0.78 to −0.14)	−0.01 (−0.43 to 0.42)	0.41 (−0.01 to 0.71)	−0.39 (−0.70 to 0.04)	−0.01 (−0.43 to 0.41)
HI_angle,mean_	−0.34 (−0.67 to 0.09)	−0.65** (−0.84 to −0.31)	0.07 (−0.37 to 0.47)	0.58** (0.21 to 0.80)	−0.52* (−0.77 to −0.12)	−0.04 (−0.45 to 0.39)
FD_Hor_	−0.28 (−0.63 to 0.16)	0.56** (0.18 to 0.79)	−0.72** (−0.87 to −0.42)	−0.42 (−0.71 to 0.00)	0.18 (−0.26 to 0.56)	0.55** (0.17 to 0.79)
FD_Ver_	−0.17 (−0.55 to 0.27)	0.68** (0.37 to 0.86)	−0.77** (−0.90 to −0.52)	0.65** (−0.84 to −0.32)	0.39 (−0.04 to 0.69)	0.45* (0.04 to 0.73)

*p < 0.05, **p < 0.01; WB, water bath; *E*_Lap_, entropy of the Laplacian-based image; FD, fractal dimension of horizontal (Hor) or vertical (Ver) structures; *E*_LBP_, entropy of grouped local binary patterns; HI_angle_, homogeneity index for orientation of local patterns; BV/TV, bone volume fraction; Conn.Dn, connectivity density; Tb.Th, trabecular thickness; Tb.Sp, trabecular separation; Tb.N, trabecular number; SMI, structure model index

Significant correlations between bone structure parameters assessed from both the original 3D *µ*CT data and its 2D projection were also obtained (Tables 3, 4, 5 and Supplementary Tables 8, 9, 10, and 11).

## Discussion

Current results for full thickness tibial bones indicate that estimates for both the subchondral bone density and structure, evaluated from 2D plain radiographs, are significantly correlated with the corresponding 3D parameters from *µ*CT.

The bone density estimated from the plain radiograph after grayscale calibration to the aluminum step wedge (GV_mmAl_) was strongly related with the bone volume fraction from *µ*CT. The correlation remained high even when the effect of soft tissue was simulated with the water bath. The water bath increased scattering and decreased the quality of the image. The mean grayscale value from the 2D coronal projection from binarized *µ*CT slices correlated also strongly with the bone volume fraction. This finding is in line with a previous study that showed a strong correlation (*r* = 0.90) between 2D density estimate and bone volume fraction from 3D *µ*CT data.^37^ Therefore, based on the current results, estimation of bone volumetric density from 2D radiographs is feasible at least when the grayscale values corresponding to the bone falls into the range of the step wedge grayscale values. In this case, aluminum thickness corresponding to the mean grayscale value of bone can be linearly interpolated. However, if the grayscale values of the bone are outside the range of the step wedge (i.e. corresponding aluminum thickness needs to be extrapolated), the method may be less reliable since the detector response of the X-ray device is usually not perfectly linear.

From the texture parameters evaluated from the 2D radiograph, especially fractal dimension of vertical structures (FD_Ver_) and mean homogeneity index for orientation of local patterns (HI_angle,mean_) were significantly related with the connectivity density and trabecular separation in 3D. This finding for the fractal dimension is consistent with an earlier study.^22^ Since the HI_angle,mean_ parameter is the mean HI value from four different directions, it is less affected by the orientation of the image. It can be hypothesized that if the image would be oriented along the trabeculae, fractal dimensions or directional homogeneity indices would correlate even more strongly with thickness and separation of trabeculae. Our results support this hypothesis since the degree of correlation varied depending on which direction the fractal dimension or homogeneity indices of local patterns were calculated. For example, HI_angle_ in the horizontal direction and FD_Ver_ in trabecular bone area were significantly related with the trabecular separation whereas HI_angle_ in the vertical direction and FD_Hor_ were less related. This is because the trabeculae were aligned more vertically than horizontally in this data set and, thus, when calculating HI_angle_, there were less variation in the orientation of adjacent local patterns in vertical direction. However, in the previous texture analysis studies of a knee joint, the images have not been oriented along the main direction of trabeculae and therefore we decided to not orient the images either. It should be noted that when calculating FD_Ver_, the structuring element was actually pointing in the horizontal direction.^20^ Therefore, FD_Ver_ and HI_angle_ in the horizontal direction are related although not directly measuring the same phenomenon.

The degrees of correlation between *µ*CT parameters and entropies (Laplacian-based or entropy of grouped patterns) were variable. One explanation for the positive correlation between entropy of grouped patterns and connectivity density is that when the bone is highly connected, more different patterns are detected in the texture analysis and eventually the entropy of patterns is therefore higher. Laplacian-based entropy might be better suited for the analysis of femoral neck, where the orientation of the trabeculae is usually clear and the ROI can be easily aligned along the trabeculae.^39^ In the current study, the Laplacians were calculated in the vertical and horizontal directions and summed together, which may have decreased the sensitivity of method for bone changes.

In our analyses, both the subchondral bone and trabecular bone VOIs contained both cortical and trabecular bone. These bone types are also superimposed in the plain radiograph and, therefore, we did not extract cortical bone layer from the *µ*CT analyses. Furthermore, the cortical bone layer is very thin at the area from which the trabecular bone VOI was extracted. When cortical bone was removed from the *µ*CT data, the results remained virtually the same (data not shown, tested with medial trabecular bone VOIs). This has also been confirmed in an earlier study in which bone volume fraction was calculated with and without cortical bone and a strong correlation was obtained (*r* = 0.73).^10^

Our results show that radiographic texture analysis may serve as a complementary method in OA diagnostics since good correlations with 3D microarchitecture of bone were obtained. This conclusion is supported by the finding that the bone density- and structure-related parameters from radiographs correlated significantly with 3D *µ*CT parameters that have been shown to change during OA. In general, osteoarthritic subchondral bone has higher bone volume fraction and trabeculae are thicker than in healthy bone.^2^,^4^,^7^–^9^,^13^ Structure model index has been reported to be lower in OA bone than in the control bone.^7^,^8^ Some studies have reported higher connectivity and increased trabecular separation but fewer trabeculae in OA bone compared to controls.^8^,^13^ However, there are also studies suggesting that in OA bone, number of trabeculae is higher and they are closer together than in controls.^2^,^4^,^7^–^9^ This discordance is likely due to the difference in anatomical sites studied and to the different stages of OA in the samples.

The degrees of correlations between radiographic parameters and 3D microarchitecture of bone were not similar in each ROI in the current study. Several factors including differences between subchondral and trabecular bone regions as well as between medial and lateral regions have influenced these variations. For example, organization of trabecular network is not similar over these regions, which might have affected the bone structural parameters as discussed earlier. Furthermore, the range in bone volume fraction was smallest in the medial trabecular bone and, therefore, the degree of correlation between the radiograph-based density and bone volume fraction might have been lower in this area. As the pooling of the ROIs might have artificially increased the significance of the correlation coefficients, the correlation coefficients for each ROI are separately shown in the Supplementary Material.

The most significant limitation of the current study is that the donors did not have any diagnosed joint disease at the time of death and, thus, only limited variation in bone density and structure was presumed. However, as the age range of the cadavers was 29–77 years, it is highly probable that some of the bones from older cadavers have actually had some osteoarthritic tissue-level changes. This assumption is further supported by the histological Mankin scores available for the cartilage-bone samples drilled from the contralateral knee^32^: the Mankin scores varied from 1 to 9 (healthy = 0, severe OA = 14) for the samples from the medial and lateral tibial plateaus. On the other hand, the variability of the 3D parameter values obtained from the *µ*CT data also suggests that the study sample was relatively heterogeneous. Consequently, actual variation in bone density and structure in this sample set has probably been higher than could be presumed for completely intact samples. Nevertheless, as the visual signs of OA were not specifically evaluated from these tibiae, this limitation remains and justifies future studies with a larger sample set including both non-OA and OA subjects to further clarify the sensitivity of the methods reported here. As second limitation, our samples did not contain soft tissue that reduces quality of the radiograph and, therefore, generalization of the methods in vivo is partially restricted. However, the effect of soft tissue was still simulated by immersing the bones into a water bath during radiography.

In conclusion, estimates for the subchondral bone density and structure, evaluated from 2D plain radiographs, were significantly correlated with the corresponding 3D parameters from *µ*CT. Therefore, evaluation of bone density and bone structure is feasible from the standard clinically available radiographs.

## Electronic supplementary material

Below is the link to the electronic supplementary material.

Supplementary material 1 (DOCX 66 kb)

## Abbreviations

2D: 2-Dimensional
3D: 3-Dimensional
CT: Computed tomography
FSA: Fractal signature analysis
LBP: Local binary patterns
*µ*CT: Micro computed tomography
OA: Osteoarthritis
ROI: Region of interest
VOI: Volume of interest
