# Supplementary material for: Correlation of Subchondral Bone Density and Structure from Plain Radiographs with Micro Computed Tomography Ex Vivo
Source: Ann Biomed Eng. 2015 Sep 14;44:1698–709. doi: 10.1007/s10439-015-1452-y (PMC4696139; doi:10.1007/s10439-015-1452-y)
Supplement: Supplementary file 1 — Supplementary material 1 (DOCX 66 kb) [file 10439_2015_1452_MOESM1_ESM.docx]

**Supplementary Tables**

**Table 6**. Mean and standard deviation (SD) of 3D parameters in each volume of interest from µCT.

| 3D parameters | Medial subchondral bone  Mean ± SD (min – max)  (*n* = 11) | Lateral subchondral bone  Mean ± SD (min – max)  (*n* = 11) | Medial trabecular bone  Mean ± SD (min – max)  (*n* = 11) | Lateral trabecular bone  Mean ± SD (min – max)  (*n* = 11) |
| --- | --- | --- | --- | --- |
| BV/TV (%) | 26.0 ± 4.7 (18.9 – 33.6) | 25.7 ± 5.8 (17.2 – 32.8) | 18.5 ± 3.1 (14.2 – 23.1) | 17.0 ± 3.5 (11.8 – 25.1) |
| Conn.Dn (1/mm^3^) | 7.50 ± 2.46 (3.82 – 11.51) | 7.30 ± 1.64 (4.68 – 9.83) | 5.33 ± 1.54 (3.27 – 8.53) | 5.16 ± 1.26 (3.14 – 6.78) |
| Tb.Th (µm) | 230 ± 25 (198 – 283) | 227 ± 30 (178 – 278) | 195 ± 22 (173 – 250) | 193 ± 24 (162 – 243) |
| Tb.Sp (µm) | 660 ± 108 (532 – 856) | 653 ± 79 (526 – 801) | 759 ± 87 (634 – 901) | 819 ± 93 (686 – 997) |
| Tb.N (1/mm) | 1.13 ± 0.17 (0.92 – 1.41) | 1.13 ± 0.15 (0.97 – 1.41) | 0.95 ± 0.13 (0.83 – 1.21) | 0.88 ± 0.12 (0.73 – 1.13) |
| SMI | 0.87 ± 0.27 (0.50 – 1.27) | 0.79 ± 0.35 (0.28 – 1.31) | 1.10 ± 0.26 (0.74 – 1.63) | 1.17 ± 0.22 (0.65 – 1.49) |

BV/TV = bone volume fraction, Conn.Dn = connectivity density, Tb.Th = trabecular thickness, Tb.Sp = trabecular separation, Tb.N = trabecular number, SMI = structure model index.

**Table 7**. Pearson correlation coefficients (95% confidence interval) between bone densities evaluated from both plain radiographs and 2D µCT projection image and BV/TV.

| Parameter | BV/TV |  | BV/TV |  | BV/TV |  | BV/TV |
| --- | --- | --- | --- | --- | --- | --- | --- |
|  | Medial subchondral bone (*n* = 11) |  | Lateral subchondral bone  (*n* = 11) |  | Medial trabecular bone  (*n* = 11) |  | Lateral trabecular bone  (*n* = 11) |
| Plain radiograph: |  |  |  |  |  |  |  |
| GV_mmAl_ | 0.79 (0.35 – 0.94)** |  | 0.84 (0.49 – 0.96)** |  | 0.46 (-0.20 – 0.83) |  | 0.76 (0.30 – 0.93)** |
| 2D µCT projection image: | |  |  |  |  |  |  |
| GV | 0.93 (0.80 – 0.99)** |  | 0.93 (0.75 – 0.98)** |  | 0.77 (0.33 – 0.94)** |  | 0.96 (0.84 – 0.99)** |

***p*<0.01, GV = mean grayscale value, GV_mmAl_ = GV converted to aluminum equivalents.

**Table 8**. Pearson correlation coefficients (95% confidence interval) between bone structure-related parameters from both plain radiographs and 2D µCT projection image and µCT parameters in medial subchondral bone ROIs (*n* = 11).

| Parameter | BV/TV | Conn.Dn | | | | Tb.Th | | | Tb.Sp. | | | Tb.N. | SMI | | |  |
| --- | --- | --- | --- | --- | --- | --- | --- | --- | --- | --- | --- | --- | --- | --- | --- | --- |
| Plain radiograph: | | |  |  | | |  | | |  | | |  | |  |  |
| E_Lap_ | 0.55  (-0.07 – 0.87) | 0.10  (-0.53 – 0.66) | | | | 0.55  (-0.07 – 0.86) | | | -0.31  (-0.77 – 0.36) | | | 0.34  (-0.35 – 0.77) | -0.41  (-0.81 – 0.25) | | |  |
| E_LBP_ | 0.14  (-0.50 – 0.68) | 0.69*  (0.15 – 0.91) | | | | -0.40  (-0.81 – 0.27) | | | -0.47*  (-0.83 – 0.18) | | | 0.50  (-0.14 – 0.85) | 0.19  (-0.46 – 0.71) | | |  |
| $\mathrm{HI}_{\mathrm{angle}}^{\mathrm{Hor}}$ | -0.10  (-0.66 – 0.53) | -0.60*  (-0.88 – 0.00) | | | | 0.42  (-0.24 – 0.81) | | | 0.57  (-0.05 – 0.87) | | | -0.41  (-0.81 – 0.25) | -0.39  (-0.80 – 0.27) | | |  |
| $\mathrm{HI}_{\mathrm{angle}}^{\mathrm{Ver}}$ | -0.72*  (-0.92 – -0.22) | -0.56  (-0.87 – 0.06) | | | | -0.12  (-0.67 – 0.52) | | | 0.82**  (0.43 – 0.95) | | | -0.76**  (-0.93 – -0.30) | 0.33  (-0.33 – 0.78) | | |  |
| HI_angle,mean_ | -0.57  (-0.87 – 0.04) | -0.69*  (-0.91 – -0.16) | | | | 0.13  (-0.51 – 0.68) | | | 0.85**  (0.51 – 0.96) | | | -0.75**  (-0.93 – -0.28) | 0.08  (-0.54 – 0.65) | | |  |
| FD_Hor_ | 0.09  (-0.54 – 0.65) | 0.53  (-0.10 – 0.86) | | | | -0.35  (-0.78 – 0.32) | | | -0.34  (-0.78 – 0.33) | | | 0.39  (-0.27 – 0.80) | 0.09  (-0.54 – 0.66) | | |  |
| FD_Ver_ | 0.02  (-0.59 – 0.61) | 0.71*  (0.19 – 0.92) | | | | -0.63*  (-0.89 – -0.05) | | | -0.60  (-0.88 – 0.00) | | | 0.45  (-0.20 – 0.83) | 0.46  (-0.19 – 0.83) | | |  |
| 2D µCT projection image: | | |  | |  | | |  | | |  | | |  | | |
| E_Lap_ | 0.69**  (0.15 – 0.91) | 0.45  (-0.21 – 0.83) | | | | 0.20  (-0.45 – 0.72) | | | -0.65*  (-0.90 – -0.08) | | | 0.67*  (0.12 – 0.91) | -0.50  (-0.85 – 0.14) | | |  |
| E_LBP_ | 0.69**  (0.14 – 0.91) | 0.79**  (0.37 – 0.94) | | | | -0.06  (-0.64 – 0.56) | | | -0.85**  (-0.96 – -0.51) | | | 0.89**  (0.61 – 0.97) | -0.28  (-0.75 – 0.39) | | |  |
| $\mathrm{HI}_{\mathrm{angle}}^{\mathrm{Hor}}$ | -0.70*  (-0.92 – -0.17) | -0.79**  (-0.94 – -0.35) | | | | 0.00  (-0.60 – 0.56) | | | 0.83**  (0.45 – 0.93) | | | -0.87**  (-0.97 – -0.41) | 0.29  (-0.38 – 0.76) | | |  |
| $\mathrm{HI}_{\mathrm{angle}}^{\mathrm{Ver}}$ | -0.69**  (-0.91 – -0.15) | -0.68**  (-0.91 – -0.14) | | | | -0.07  (-0.64 – 0.60) | | | 0.75**  (0.27 – 0.93) | | | -0.81**  (-0.95 – -0.41) | 0.34  (-0.32 – 0.78) | | |  |
| HI_angle,mean_ | -0.69*  (-0.91 – -0.15) | -0.78**  (-0.94 – -0.35) | | | | 0.01  (-0.60 – 0.60) | | | 0.82**  (0.44 – 0.95) | | | -0.86**  (-0.96 – -0.55) | 0.27  (-0.39 – 0.75) | | |  |
| FD_Hor_ | -0.26  (-0.74 – 0.41) | 0.43  (-0.23 – 0.82) | | | | -0.65*  (-0.90 – -0.07) | | | -0.11  (-0.67 – 0.53) | | | 0.17  (-0.48 – 0.70) | 0.37  (-0.30 – 0.79) | | |  |
| FD_Ver_ | 0.11  (-0.53 – 0.66) | 0.83**  (0.46 – 0.95) | | | | -0.68*  (-0.91 – -0.13) | | | -0.62*  (-0.89 – -0.03) | | | 0.60  (0.00 – 0.88) | 0.33  (-0.34 – 0.78) | | |  |

**p*<0.05, ***p*<0.01, E_Lap_ = entropy of the Laplacian-based image, FD = fractal dimension of horizontal (Hor) or vertical (Ver) structures, E_LBP_ = entropy of grouped local binary patterns, HI_angle_ = homogeneity index for orientation of local patterns, BV/TV = bone volume fraction, Conn.Dn = connectivity density, Tb.Th = trabecular thickness, Tb.Sp = trabecular separation, Tb.N = trabecular number, SMI = structure model index.

**Table 9**. Pearson correlation coefficients (95% confidence interval) between bone structure-related parameters from both plain radiographs and 2D µCT projection image and µCT parameters in lateral subchondral bone ROIs (*n* = 11).

| Parameter | BV/TV | Conn.Dn | | | Tb.Th | | Tb.Sp. | | Tb.N. | SMI |
| --- | --- | --- | --- | --- | --- | --- | --- | --- | --- | --- |
| Plain radiograph: | |  | | |  | |  | |  |  |
| E_Lap_ | 0.68*  (0.13 – 0.91) | 0.40  (-0.27 – 0.80) | | | 0.53  (-0.10 – 0.86) | | -0.68*  (-0.91 – -0.13) | | 0.65*  (0.08 – 0.90) | -0.63*  (-0.89 – -0.04) |
| E_LBP_ | 0.48  (-0.17 – 0.84) | 0.23  (-0.43 – 0.73) | | | 0.54  (-0.10 – 0.86) | | -0.45  (-0.83 – 0.20) | | 0.31  (-0.35 – 0.77) | -0.34  (-0.78 – 0.33) |
| $\mathrm{HI}_{\mathrm{angle}}^{\mathrm{Hor}}$ | -0.23  (-0.73 – 0.43) | -0.27  (-0.75 – 0.39) | | | 0.01  (-0.59 – 0.61) | | 0.24  (-0.42 – 0.74) | | -0.41  (-0.81 – 0.25) | 0.27  (-0.39 – 0.75) |
| $\mathrm{HI}_{\mathrm{angle}}^{\mathrm{Ver}}$ | -0.43  (-0.82 – 0.23) | -0.10  (-0.66 – 0.53) | | | -0.59  (-0.88 – 0.02) | | 0.34  (-0.33 – 0.78) | | -0.19  (-0.71 – 0.47) | 0.26  (-0.40 – 0.74) |
| HI_angle,mean_ | -0.45  (-0.83 – 0.20) | -0.27  (-0.75 – 0.39) | | | 0.43  (-0.82 – 0.23) | | 0.42  (-0.24 – 0.81) | | -0.39  (-0.80 – 0.28) | 0.35  (-0.31 – 0.79) |
| FD_Hor_ | -0.12  (-0.67 – 0.52) | 0.25  (-0.42 – 0.74) | | | -0.38  (-0.80 – 0.29) | | -0.03  (-0.62 – 0.58) | | 0.14  (-0.51 – 0.68) | 0.08  (-0.55 – 0.65) |
| FD_Ver_ | 0.41  (-0.25 – 0.81) | 0.34  (-0.33 – 0.78) | | | 0.29  (-0.38 – 0.76) | | -0.47  (-0.84 – 0.18) | | 0.44  (-0.22 – 0.82) | -0.37  (-0.79 – 0.30) |
| 2D µCT projection image: | | |  |  | |  | |  | |  |
| E_Lap_ | 0.48  (-0.17 – 0.84) | 0.30  (-0.37 – 0.76) | | | 0.41  (-0.25 – 0.81) | | -0.47  (-0.83 – 0.19) | | 0.42  (-0.24 – 0.82) | -0.37  (-0.79 – 0.30) |
| E_LBP_ | 0.68*  (0.13 – 0.91) | 0.72*  (0.20 – 0.92) | | | 0.43  (-0.23 – 0.82) | | -0.80**  (-0.95 – -0.39) | | 0.76**  (-0.29 – 0.93) | -0.54  (-0.86 – 0.08) |
| $\mathrm{HI}_{\mathrm{angle}}^{\mathrm{Hor}}$ | -0.56  (-0.87 – 0.06) | -0.74**  (-0.93 – -0.26) | | | -0.23  (-0.73 – 0.43) | | 0.75**  (0.26 – 0.93) | | -0.74**  (-0.93 – -0.25) | 0.48  (-0.17 – 0.84) |
| $\mathrm{HI}_{\mathrm{angle}}^{\mathrm{Ver}}$ | -0.60  (-0.88 – 0.00) | -0.68*  (-0.91 – -0.14) | | | -0.33  (-0.78 – 0.34) | | 0.73*  (0.23 – 0.92) | | -0.71*  (-0.92 – -0.19) | 0.51  (-0.13 – 0.85) |
| HI_angle,mean_ | -0.57  (-0.87 – 0.05) | -0.75**  (-0.93 – -0.27) | | | -0.25  (-0.74 – 0.42) | | 0.75**  (0.28 – 0.93) | | -0.73*  (-0.92 – -0.23) | 0.47  (-0.18 – 0.83) |
| FD_Hor_ | -0.29  (-0.76 – 0.38) | 0.35  (-0.32 – 0.78) | | | -0.54  (-0.86 – 0.08) | | -0.01  (-0.60 – 0.60) | | 0.02  (-0.59 – 0.61) | 0.27  (-0.39 – 0.75) |
| FD_Ver_ | -0.05  (-0.63 – 0.57) | 0.74**  (0.24 – 0.93) | | | -0.43  (-0.82 – 0.23) | | -0.45  (-0.83 – 0.21) | | 0.32  (-0.35 – 0.77) | 0.16  (-0.49 – 0.69) |

**p*<0.05, ***p*<0.01, E_Lap_ = entropy of the Laplacian-based image, FD = fractal dimension of horizontal (Hor) or vertical (Ver) structures, E_LBP_ = entropy of grouped local binary patterns, HI_angle_ = homogeneity index for orientation of local patterns, BV/TV = bone volume fraction, Conn.Dn = connectivity density, Tb.Th = trabecular thickness, Tb.Sp = trabecular separation, Tb.N = trabecular number, SMI = structure model index.

**Table 10**. Pearson correlation coefficients (95% confidence interval) between bone structure-related parameters from both plain radiographs and 2D µCT projection image and µCT parameters in medial trabecular bone ROIs (*n* = 11).

| Parameter | BV/TV | Conn.Dn | | | | Tb.Th | | | Tb.Sp. | | | Tb.N. | SMI | | |  |
| --- | --- | --- | --- | --- | --- | --- | --- | --- | --- | --- | --- | --- | --- | --- | --- | --- |
| Plain radiograph: | | |  |  | | |  | | |  | | |  | |  |  |
| E_Lap_ | 0.17  (-0.48 – 0.70) | -0.23  (-0.73 – 0.43) | | | | 0.44  (-0.22 – 0.82) | | | 0.27  (-0.40 – 0.75) | | | -0.10  (-0.66 – 0.53) | -0.18  (-0.71 – 0.47) | | |  |
| E_LBP_ | 0.25  (-0.41 – 0.74) | 0.66*  (0.09 – 0.90) | | | | -0.24  (-0.74 – 0.42) | | | -0.71*  (-0.92 – -0.19) | | | 0.52  (-0.12 – 0.85) | 0.18  (-0.47 – 0.70) | | |  |
| $\mathrm{HI}_{\mathrm{angle}}^{\mathrm{Hor}}$ | -0.13  (-0.67 – 0.51) | -0.92**  (-0.98 – -0.70) | | | | 0.63*  (0.04 – 0.89) | | | 0.88**  (0.58 – 0.97) | | | -0.67*  (-0.91 – -0.12) | -0.39  (-0.80 – 0.28) | | |  |
| $\mathrm{HI}_{\mathrm{angle}}^{\mathrm{Ver}}$ | -0.46  (-0.83 – 0.19) | -0.74**  (-0.93 – -0.25) | | | | -0.14  (-0.50 – 0.68) | | | 0.84**  (0.47 – 0.96) | | | -0.71*  (-0.92 – -0.19) | -0.05  (-0.63 – 0.57) | | |  |
| HI_angle,mean_ | -0.33  (-0.78 – 0.33) | -0.88**  (-0.97 – -0.58) | | | | 0.39  (-0.27 – 0.80) | | | 0.91**  (0.68 – 0.98) | | | -0.75**  (-0.93 – -0.26) | -0.21  (-0.72 – 0.45) | | |  |
| FD_Hor_ | -0.06  (-0.64 – 0.56) | 0.37  (-0.29 – 0.80) | | | | -0.42  (-0.81 – 0.25) | | | -0.35  (-0.78 – 0.32) | | | 0.25  (-0.41 – 0.74) | 0.21  (-0.45 – 0.72) | | |  |
| FD_Ver_ | -0.05  (-0.63 – 0.57) | 0.72*  (0.21 – 0.92) | | | | -0.73*  (-0.93 – -0.23) | | | -0.71*  (-0.92 – -0.20) | | | 0.50  (-0.14 – 0.85) | 0.36  (-0.31 – 0.79) | | |  |
| 2D µCT projection image: | | |  | |  | | |  | | |  | | |  | | |
| E_Lap_ | 0.50  (-0.14 – 0.85) | -0.05  (-0.63 – 0.56) | | | | 0.57  (-0.05 – 0.87) | | | 0.07  (-0.56 – 0.64) | | | 0.21  (-0.45 – 0.72) | -0.51  (-0.85 – 0.13) | | |  |
| E_LBP_ | 0.49  (-0.15 – 0.84) | 0.65*  (0.08 – 0.90) | | | | -0.01  (-0.60 – 0.60) | | | -0.77**  (-0.94 – -0.31) | | | 0.64*  (0.07 – 0.90) | 0.08  (-0.55 – 0.65) | | |  |
| $\mathrm{HI}_{\mathrm{angle}}^{\mathrm{Hor}}$ | -0.38  (-0.80 – 0.29) | -0.67*  (-0.90 – -0.11) | | | | 0.15  (-0.49 – 0.69) | | | 0.80**  (0.39 – 0.95) | | | -0.61*  (-0.89 – -0.02) | -0.25  (-0.74 – 0.41) | | |  |
| $\mathrm{HI}_{\mathrm{angle}}^{\mathrm{Ver}}$ | -0.41  (-0.81 – 0.25) | -0.59  (-0.88 – 0.01) | | | | 0.03  (-0.58 – 0.62) | | | 0.73*  (0.24 – 0.93) | | | -0.56  (-0.87 – 0.06) | -0.18  (-0.70 – 0.47) | | |  |
| HI_angle,mean_ | -0.38  (-0.80 – 0.28) | -0.64*  (-0.90 – -0.06) | | | | -0.11  (-0.53 – 0.66) | | | 0.78**  (0.34 – 0.94) | | | -0.59  (-0.88 – 0.02) | -0.24  (-0.73 – 0.42) | | |  |
| FD_Hor_ | -0.19  (-0.71 – 0.46) | 0.56  (-0.06 – 0.87) | | | | -0.69*  (-0.91 – -0.14) | | | -0.55  (-0.87 – 0.07) | | | 0.31  (-0.36 – 0.77) | 0.60  (0.00 – 0.88) | | |  |
| FD_Ver_ | -0.20  (-0.72 – 0.45) | 0.75**  (0.27 – 0.93) | | | | -0.85**  (-0.96 – -0.52) | | | -0.70**  (-0.91 – -0.17) | | | 0.42  (-0.24 – 0.82) | 0.58  (-0.03 – 0.87) | | |  |

**p*<0.05, ***p*<0.01, E_Lap_ = entropy of the Laplacian-based image, FD = fractal dimension of horizontal (Hor) or vertical (Ver) structures, E_LBP_ = entropy of grouped local binary patterns, HI_angle_ = homogeneity index for orientation of local patterns, BV/TV = bone volume fraction, Conn.Dn = connectivity density, Tb.Th = trabecular thickness, Tb.Sp = trabecular separation, Tb.N = trabecular number, SMI = structure model index.

**Table 11**. Pearson correlation coefficients (95% confidence interval) between bone structure-related parameters from both plain radiographs and 2D µCT projection image and µCT parameters in lateral trabecular bone ROIs (*n* = 11).

| Parameter | BV/TV | Conn.Dn | | | Tb.Th | | Tb.Sp. | | Tb.N. | SMI |
| --- | --- | --- | --- | --- | --- | --- | --- | --- | --- | --- |
| Plain radiograph: | |  | | |  | |  | |  |  |
| E_Lap_ | 0.30  (-0.36 – 0.76) | 0.06  (-0.56 – 0.64) | | | 0.26  (-0.40 – 0.74) | | -0.21  (-0.72 – 0.45) | | 0.25  (-0.41 – 0.74) | -0.35  (-0.79 – 0.31) |
| E_LBP_ | 0.36  (-0.30 – 0.79) | 0.30  (-0.36 – 0.76) | | | 0.13  (-0.51 – 0.68) | | -0.28  (-0.75 – 0.38) | | 0.42  (-0.24 – 0.81) | -0.42  (-0.82 – 0.24) |
| $\mathrm{HI}_{\mathrm{angle}}^{\mathrm{Hor}}$ | 0.06  (-0.56 – 0.64) | -0.69*  (-0.91 – -0.15) | | | 0.57  (-0.05 – 0.87) | | 0.61*  (0.02 – 0.89) | | -0.38  (-0.80 – 0.28) | -0.31  (-0.77 – 0.36) |
| $\mathrm{HI}_{\mathrm{angle}}^{\mathrm{Ver}}$ | -0.33  (-0.78 – 0.34) | -0.59  (-0.88 – 0.02) | | | 0.16  (-0.49 – 0.69) | | 0.47  (-0.18 – 0.84) | | -0.59  (-0.88 – 0.02) | 0.37  (-0.29 – 0.79) |
| HI_angle,mean_ | -0.01  (-0.60 – 0.60) | -0.79**  (-0.94 – -0.37) | | | 0.59  (-0.02 – 0.88) | | 0.68*  (0.13 – 0.91) | | -0.49  (-0.84 – 0.15) | -0.20  (-0.71 – 0.45) |
| FD_Hor_ | -0.63*  (-0.89 – -0.04) | -0.20  (-0.71 – 0.46) | | | -0.52  (-0.85 – 0.11) | | 0.24  (-0.42 – 0.74) | | -0.51  (-0.85 – 0.12) | 0.60*  (0.00 – 0.88) |
| FD_Ver_ | -0.30  (-0.76 – 0.36) | 0.59  (-0.02 – 0.88) | | | -0.72*  (-0.92 – -0.21) | | -0.52  (-0.85 – 0.11) | | 0.14  (-0.50 – 0.68) | 0.56  (-0.05 – 0.87) |
| 2D µCT projection image: | | |  |  | |  | |  | |  |
| E_Lap_ | 0.12  (-0.52 – 0.67) | -0.35  (-0.79 – 0.31) | | | 0.46  (-0.20 – 0.83) | | 0.39  (-0.28 – 0.80) | | -0.19  (-0.71 – 0.46) | -0.20  (-0.71 – 0.46) |
| E_LBP_ | 0.63*  (0.05 – 0.89) | 0.68*  (0.14 – 0.91) | | | 0.23  (-0.43 – 0.73) | | -0.70*  (-0.92 – -0.17) | | 0.77**  (0.32 – 0.94) | -0.40  (-0.80 – 0.27) |
| $\mathrm{HI}_{\mathrm{angle}}^{\mathrm{Hor}}$ | -0.51  (-0.85 – 0.12) | -0.83**  (-0.95 – -0.45) | | | 0.04  (-0.58 – 0.62) | | 0.80**  (0.38 – 0.95) | | -0.80**  (-0.95 – -0.39) | 0.31  (-0.35 – 0.77) |
| $\mathrm{HI}_{\mathrm{angle}}^{\mathrm{Ver}}$ | -0.59  (-0.88 – 0.01) | -0.76**  (-0.93 – -0.29) | | | -0.12  (-0.67 – 0.51) | | 0.74**  (0.25 – 0.93) | | -0.79**  (-0.94 – -0.36) | 0.40  (-0.27 – 0.80) |
| HI_angle,mean_ | -0.54  (-0.86 – 0.09) | -0.81**  (-0.95 – -0.42) | | | -0.02  (-0.61 – 0.59) | | 0.79**  (0.35 – 0.94) | | -0.80**  (-0.95 – -0.39) | 0.33  (-0.33 – 0.78) |
| FD_Hor_ | -0.28  (-0.75 – 0.39) | 0.65*  (0.08 – 0.90) | | | -0.76**  (-0.93 – -0.30) | | -0.54  (-0.86 – 0.09) | | 0.22  (-0.44 – 0.72) | 0.47  (-0.18 – 0.84) |
| FD_Ver_ | -0.34  (-0.78 – 0.33) | 0.65*  (0.08 – 0.90) | | | -0.85**  (-0.96 – -0.51) | | -0.52  (-0.85 – 0.12) | | 0.20  (-0.45 – 0.71) | 0.50  (-0.15 – 0.85) |

**p*<0.05, ***p*<0.01, E_Lap_ = entropy of the Laplacian-based image, FD = fractal dimension of horizontal (Hor) or vertical (Ver) structures, E_LBP_ = entropy of grouped local binary patterns, HI_angle_ = homogeneity index for orientation of local patterns, BV/TV = bone volume fraction, Conn.Dn = connectivity density, Tb.Th = trabecular thickness, Tb.Sp = trabecular separation, Tb.N = trabecular number, SMI = structure model index.
